# A New Flavonoid Glycoside from the Stem Bark of *Albizia saponaria*: Isolation, Structural Elucidation, and In Silico Evaluation as a Potent α-Glucosidase Inhibitor

**DOI:** 10.3390/ph19091391

**Published:** 2026-09-02

**Authors:** Emma Julin Pongoh, Rymond Jusuf Rumampuk

**Affiliations:** Department of Chemistry, Manado State University, Tondano 95618, North Sulawesi, Indonesia; rymondrumampuk@unima.ac.id

**Keywords:** *Albizia saponaria*, flavonoid glycoside, structural elucidation, ADMET, molecular docking, α-glucosidase inhibitor

## Abstract

**Background/Objectives**: In the search for potent non-sugar α-glucosidase inhibitors with improved safety profiles, a novel flavonoid glycoside was isolated for the first time from the stem bark of *Albizia saponaria* (Fabaceae). The objective of this study was to elucidate its chemical structure and evaluate its therapeutic potential as an anti-hyperglycemic agent compared to known related flavonoids and a standard clinical drug. **Methods**: Comprehensive structural elucidation was performed using high-resolution mass spectrometry and multidimensional 1D/2D NMR (^1^H, ^13^C, HSQC-DEPT, COSY, and CIGAR). To assess its inhibitory efficacy and pharmacokinetic profiles, an in silico comparative study was conducted against a database of related flavonoids (Quercitrin, Hyperoside, and Isoquercitrin) and the clinical drug Acarbose. This involved molecular docking simulations against human intestinal maltase-glucoamylase (PDB ID: 3TOP) alongside integrated ADMET modeling and toxicological screening. **Results**: The compound was successfully identified as 4′,7-dihydroxyflavan-3′-*O*-*β*-D-glucoside (**1**). Molecular docking revealed that Compound **1** exhibited a superior predicted binding affinity of −9.5 kcal/mol, outperforming Quercitrin (−9.3 kcal/mol), Hyperoside (−8.3 kcal/mol), Isoquercitrin (−7.9 kcal/mol), and Acarbose (−7.2 kcal/mol). This strong thermodynamic stability is driven by a robust conventional hydrogen-bonding network with key active site residues (Arg1377, Gln1372, and Gly1365), successfully overriding a localized electrostatic strain at Asp1279. Furthermore, ADMET modeling demonstrated a highly desirable local pharmacokinetic framework; its low Caco-2 permeability (−6.432) and low human intestinal absorption (HIA = 0.120) favor targeted luminal retention in the gastrointestinal tract, mirroring Acarbose while minimizing systemic exposure. Crucially, toxicological screening unveiled a significant safety advantage for Compound **1**, marked by negligible CYP3A4 interaction (0.004) and a remarkably low risk of Drug-Induced Liver Injury (DILI = 0.213) compared to the high-risk hepatotoxic profile of Acarbose (DILI = 0.882) and the reference flavonoids (DILI > 0.69). **Conclusions**: These predictive findings establish Compound **1** as a highly promising, low-toxicity natural scaffold for anti-hyperglycemic drug development. Its superior binding affinity and minimized hepatotoxicity risk warrant subsequent in vitro and in vivo functional validation.

## 1. Introduction

The genus *Albizia* (Fabaceae) comprises approximately 150 species widely distributed throughout tropical regions [1,2,3]. In Indonesia, 18 *Albizia* species have been identified, including *A. saponaria*, which is now categorized as a rare plant. Phytochemical screenings of *A. saponaria* stem bark have revealed the presence of alkaloids, tannins, saponins, phenols, and flavonoids, showcasing diverse biological properties such as anti-dandruff, antimicrobial, and strong antioxidant activities [4,5,6,7]. Flavonoids represent a major group of bioactive constituents within the *Albizia* genus, with various glycoside derivatives—such as quercitrin, isoquercitrin, and hyperoside—previously isolated from related species like *A. julibrissin*, *A. lebbeck*, and *A. amara* [8,9,10,11]. These compounds exhibit crucial pharmacological profiles, including anti-inflammatory and sedative effects. Given that flavonoid frameworks possess extensive structural diversity even within the same species, investigating the stem bark of *A. saponaria* offers a promising opportunity to discover novel scaffolds with distinct biological potentials.

Structurally diverse natural compounds play a pivotal role in drug discovery due to their unique molecular complexity. Plant-derived flavonoids, in particular, have been extensively reported to demonstrate potent antidiabetic effects through various molecular mechanisms [12]. Structurally, the presence of specific hydroxyl groups and sugar moieties on the flavonoid core contributes significantly to these antidiabetic properties by stimulating β-cell proliferation, enhancing insulin secretion, mitigating apoptosis, and modulating hepatic glucose metabolism [13]. Managing postprandial hyperglycemia via the inhibition of carbohydrate-hydrolyzing enzymes, particularly intestinal α-glucosidase, remains one of the most effective therapeutic strategies for Type 2 Diabetes Mellitus (T2DM). In the digestive tract, human intestinal maltase-glucoamylase (MGAM) is a key enzyme responsible for final starch digestion. Although commercial α-glucosidase inhibitors (AGIs) like Acarbose are widely used clinically, their flexibility imposes a heavy entropic penalty during enzyme binding, and their long-term utility is frequently limited by severe gastrointestinal side effects resulting from the fermentation of undigested carbohydrates. Consequently, there is an urgent need to discover novel, structurally rigid, non-sugar natural scaffolds that can selectively block MGAM within the intestinal lumen with fewer adverse effects.

To advance the bioprospecting of this rare species, we conducted a systematic phytochemical investigation of the stem bark of *A. saponaria*. Utilizing a combination of gel filtration and reversed-phase high-performance liquid chromatography (RP-HPLC), we successfully isolated a new flavonoid glycoside, identified as 4′,7-dihydroxyflavan-3′-*O*-*β*-D-glucoside (**1**). In this study, we report its extraction, comprehensive structural elucidation via high-resolution mass spectrometry and multidimensional 1D/2D NMR techniques (1H-1H COSY, HSQC, and CIGAR), and initial pharmacokinetic screening through in silico ADMET modeling. Furthermore, flexible molecular docking simulations against the crystal structure of human maltase-glucoamylase (PDB ID: 3TOP) were performed to predict its potential binding modes at the atomic level. The target enzyme 3TOP, located in the brush border of the small intestine, serves as the most relevant therapeutic target to evaluate whether the *β*-D-glucoside group and flavan core of Compound **1** can mimic natural carbohydrate substrates to locally inhibit maltose hydrolysis in a competitive manner.

## 2. Results

### 2.1. Isolation and Structure Elucidation of ***1***

The molecular weight of **1** was measured using electrospray ionization-mass spectrometry (ESI-MS) with positive ionization and high-resolution ESI-MS (HR-ESIMS) using negative ionization. The ESI-MS spectrum of **1** with positive ionization showed a quasi-molecular ion at m/z 421 (M + H)^+^, and the HR-ESIMS spectrum with negative ionization of this flavonoid glycoside showed a quasi-molecular ion at m/z 419.1360 (M−H)^−^, which matches the molecular formula C_21_H_24_O_9_.

Structure determination was done using one- and two-dimensional NMR techniques at 500 MHz for proton and 125 MHz for carbon. The three two-dimensional techniques used were H-H COSY, HSQC-DEPT, and CIGAR. HSQC gives information about the bond correlation between protons and carbons, DEPT shows the type of carbon (CH, CH_2_, and CH_3_), while CIGAR provides info about long-range correlations (usually two or three bonds) between protons and carbons. The ^1^H and ^13^C-NMR spectra of compound **1** are listed in Table 1.

### 2.2. Molecular Docking of Compound ***1*** Against Human Maltase-Glucoamylase (PDB ID: 3TOP)

The molecular docking simulation was successfully executed to evaluate the bind-ing capacity of the newly isolated compound **1** (4′,7-dihydroxyflavan-3′-*O*-*β*-D-glucoside) inside the catalytic domain of human maltase-glucoamylase (PDB ID: 3TOP) [14]. Before docking the test compound, method validation through redocking using the crystal’s native ligand (Acarbose) yielded a Root Mean Square Deviation (RMSD) value of 0.553 Å. The computational screening revealed that compound **1** achieved a remarkable binding affinity energy (ΔG) of −9.5 kcal/mol. Under identical simulation conditions, the reference standard drug, Acarbose, displayed an average binding energy score ranging from −6.5 to −8.2 kcal/mol [15,16].

Analysis of the optimal conformation pose showed that the structural layout of compound **1** fits tightly inside the active binding pocket. The inter-molecular interaction profile is characterized by several distinct interaction types:

(a) Conventional Hydrogen Bonds: Compound **1** established six strong, short-distance conventional hydrogen bonds anchored by its hydrophilic β-D-glucoside moiety and core hydroxyl groups. These specific links involved the amino acid residues ARG A1377, GLN A1372, and GLY A1365.
(b) Hydrophobic Engagements: The aromatic rings (Ring A and Ring B) of the flavan core formed highly stable π-π T-shaped interactions with surrounding pocket-lining aromatic residues. Additionally, multiple π-alkyl interactions were detected, bind-ing the aliphatic clusters near the non-polar sub-pocket.
(c) van der Waals Forces: A comprehensive cluster of weak electrostatic surface forces enveloped the peripheral atoms of the flavan core framework, indicating high structural complementarity.
(d) Steric Constraints: The docking interaction landscape also revealed a localized electrostatic penalty classified as an unfavorable donor-donor interaction, caused by a close-distance overlap between a hydroxyl hydrogen on the ligand and a protonated donor atom of an adjacent residue.

### 2.3. Pharmacokinetic and Toxicity Profile Analysis In Silico (ADMET) of ***1***

Evaluation of the absorption, distribution, metabolism, excretion, and toxicity (ADMET) properties of Compound **1** was done using the AI-based platforms ADMETlab 3.0 [17,18] and SwissADME [19], by looking at radar charts and Boiled Egg plots [20]. This computational approach helps predict whether a molecule can work as an oral drug and how safe its structure is before conducting lab tests. Table 2 shows a summary of the main prediction results.

## 3. Discussion

### 3.1. Structure Elucidation of ***1***

The NMR spectrum of **1**, shown in Table 1, indicates twelve aromatic carbon signals and six proton signals in the aromatic region, with chemical shifts at δ 7.30 (s), 7.24 (*d*, *J* = 8.1 Hz), 7.07 (*d*, *J* = 8.1 Hz), 6.85 (*d*, *J* = 8.3 Hz), 6.31 (*d*, *J* = 8.3 Hz), and 6.27 (s). The spectrum also shows two methylene proton signals at δ 2.82 and 2.63, which HSQC correlates with carbons at δ 25.0, and methylene protons at δ 2.13 and 1.99, which HSQC correlates with a carbon at δ 30.9, as well as a methine proton at δ 4.97, which HSQC correlates with a carbon at δ 78.4. These signals show that compound **1** is a flavonoid with a flavan core. Besides the aromatic proton signals, the NMR spectrum of **1** also shows the presence of protons and carbons from the sugar unit, with δ 3.29–4.86 for protons and δ 62.4–104.1 for carbons. The signals at δ 4.86 and 104.1 indicate the anomeric proton and carbon (protons and carbon from the sugar), but the proton multiplicity can’t be read because it overlaps with the solvent signal. Their anomeric proton signals are also supported by correlations seen in the HSQC spectrum.

The proton signal at δ 4.86 is one-bond correlated with the anomeric carbon at δ 104.1. One signal from the proton and anomeric carbon indicates that there is only one sugar unit in compound **1**. Two proton signals at δ 3.50 and 3.85 that correlate with carbon at δ 62.4 indicate the proton and carbon from the hydroxy methylene (CH_2_OH) on the sugar part. It can be concluded that the sugar unit in **1** is a hexose sugar. So, **1** is a flavonoid glycoside.

Next, we figured out the structure of **1** by analyzing correlated two-dimensional spectra (HH-COSY, HSQC-DEPT, CIGAR). The HSQC spectrum of **1** showed a one-bond correlation between the proton at δ 4.97 and the carbon at δ 78.4, which was further assigned as the proton and carbon at position 2 [21,22]. The proton signal at δ 4.97 also showed a long-range correlation (CIGAR) with the carbon at δ 25.0 (C-4), which in turn was correlated in HSQC with two protons at δ 2.82 and 2.63. Additionally, the proton at C-4 showed a CIGAR correlation with the carbon at δ 30.9 (C-3), which is attached to two protons at δ 2.13 and 1.99. CIGAR correlations were also observed between the proton at C-4 (δ 6.31) and the carbons at δ 157.90 (C-8a) and 114.7 (C-4a).

**Figure 1:**
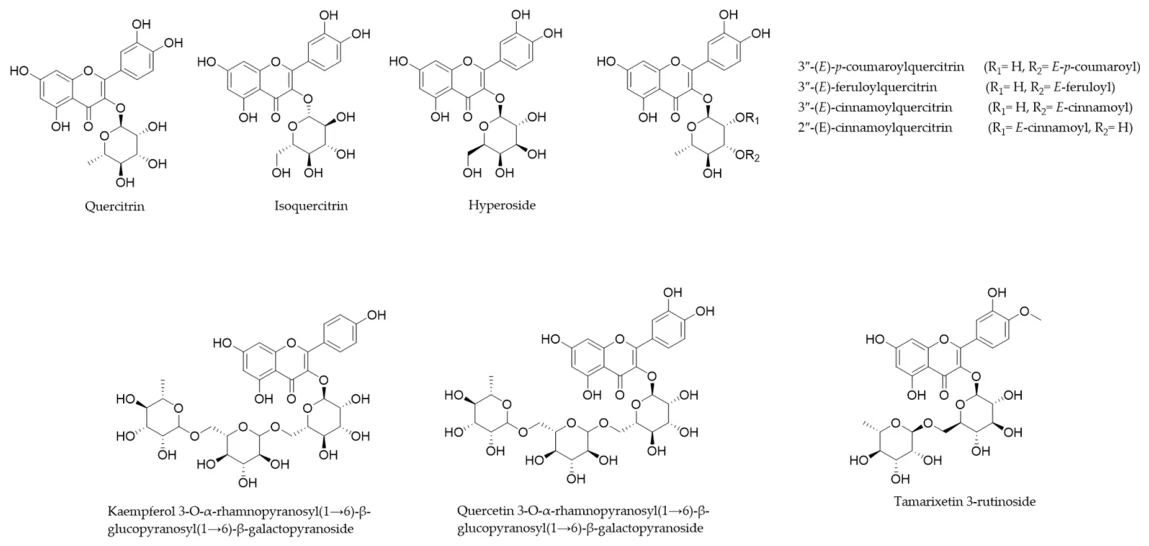
Selected flavonoid glycosides from the genus of *Albizia*.

Carbon at δ 25.0 (C-4) showed CIGAR correlation with the proton at δ 6.85 (H-5), and H-5 HSQC correlated with carbon at δ 130.9 (C-5). The proton at δ 6.85 (H-5) in the CIGAR spectrum showed correlation with the carbons at δ 157.93 and 157.90 (C-8a & C-7). HH-COSY correlation was observed between H-5 (δ 6.85) and H-6 (δ 6.31), and H-6 HSQC correlated with carbon at δ 109.3 (C-6). The proton at δ 6.31 (H-6) in the CIGAR spectrum correlated with carbon at δ 157.93 (C-7). Carbons at δ 109.3 (C-6) and δ 157.93 (C-7) each showed CIGAR correlation with the proton at δ 6.27 (H-8). Additionally, the proton signal at δ 6.27 (H-8) HSQC correlated with carbon δ 104.1 (C-8), and H-8 showed CIGAR correlation with carbons at δ 157.9 (C-8a) and 114.7 (C-4a). Based on these correlations, which are illustrated in Figure 1, we could assign the positions of protons and carbons on rings A and C. The signal C-7 at δ 157.93 indicated the presence of a paramagnetic shift due to the hydroxyl substituent, so the hydroxy group on ring A is positioned at C-7.

The determination of proton and carbon positions on ring B started with the proton at δ 7.29 (H-2′), which is HSQC correlated with the carbon at δ 118.5 (C-2′). CIGAR correlation from H-2′ was observed with carbons at δ 78.4 (C-2), 149.3 (C-3′), and 122.4 (C-4′). The C-4′ signal then HSQC correlated with the proton at δ 7.07 (H-4′), and H-4′ is CIGAR correlated with carbons at δ 118.5 (C-2′) and 149.1 (C-6′). The H-4′ signal also HH-COSY correlated with the proton at δ 7.24 (H-5′), and then the H-5′ signal HSQC correlated with carbons at δ 120.6 (C-5′) and 140.0 (C-1′). The δ 140.0 signal was set as the signal from C-1′, supported by CIGAR correlation from the proton at δ 4.97 (H-2) in ring C. The paramagnetic chemical shifts from C-3′ (149.3) and C-6′ (149.1) indicated that both carbons are oxygenated.

**Figure 2:**
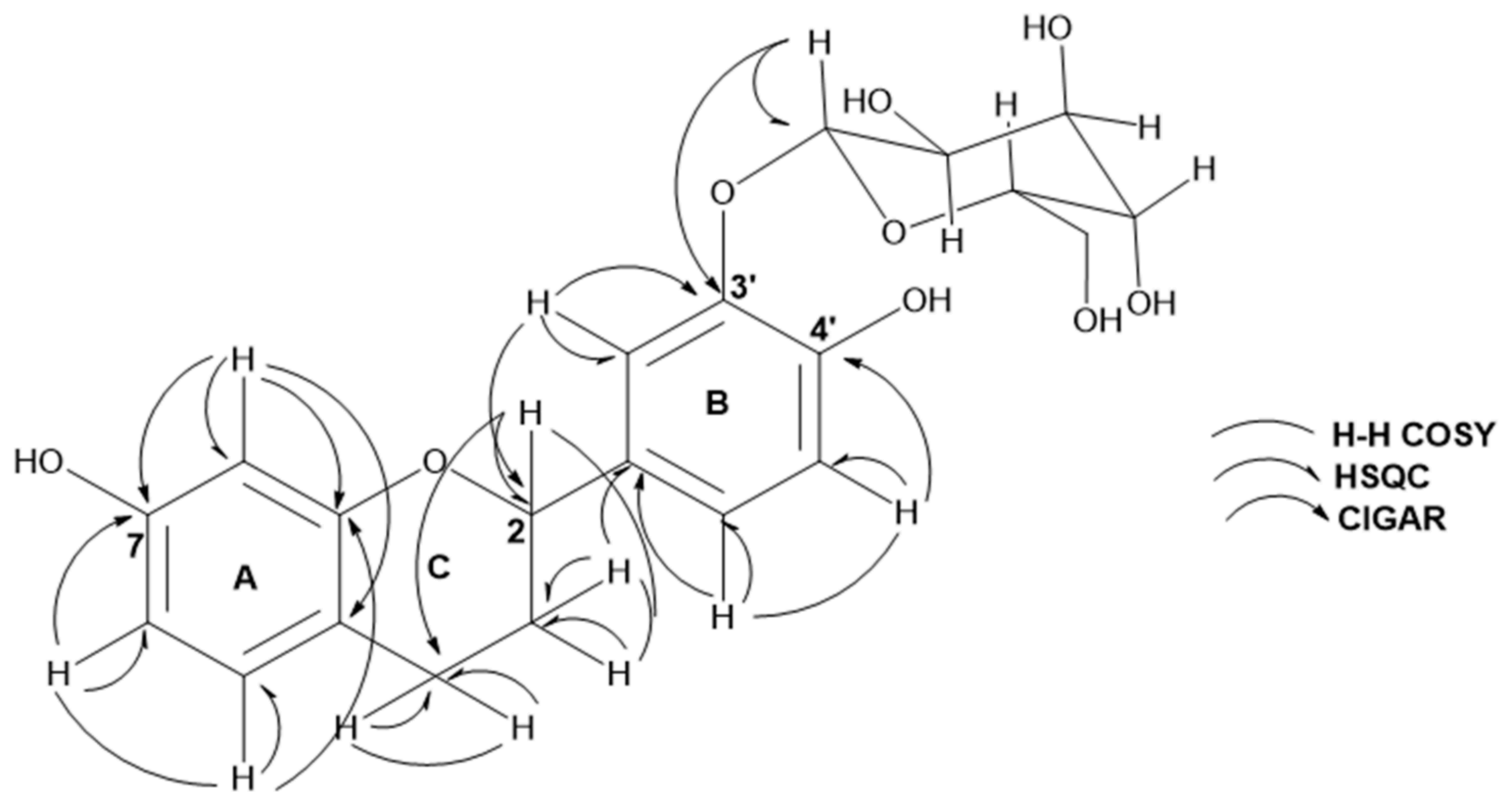
Structure of **1** (4′,7-dihydroxyflavan-3′-*O*-*β*-D-glucoside), with key correlations in H-H COSY, HSQC, and CIGAR.

Determination of the hexose sugar unit started from the anomeric proton signal. The anomeric proton (H″) showed an HH-COSY correlation with the proton at δ 3.48 (H-2″) and is HSQC correlated with the carbon at δ 75.1 (C-2). The multiplicity of H-2″ is a doublet of doublets (*J* = 8.5; 16.5 Hz), which indicates a trans-diaxial type of coupling between H-2″–H-1″ and H-2″–H-3″. Based on the coupling constant of H-2″, it was also determined that H-1″ is axial, so the orientation of the glycosidic bond in the sugar unit is β-glycosidic. From the NMR data analysis, it can be concluded that the sugar unit of **1** is D-glucose. Determination of the sugar unit position on the flavan core was carried out using the CIGAR technique. The CIGAR spectrum showed a correlation between the anomeric proton (H-1″) and the carbon at δ 149.3 (C-3′), so it can be determined that the sugar unit is attached to the C-3′ of the flavan core. Its structure is shown in Figure 2. A quick search on SciFinder Scholar shows that **1** was first isolated from the stem bark of *A. saponaria*.

### 3.2. In Silico Docking Study of ***1*** with α-Glucosidase

The in silico docking score provides a semi-empirical estimation of binding affinity to evaluate the predicted stability of a ligand–receptor complex, where a more negative score suggests a potentially favorable and stable binding conformation. To gain a deeper, comprehensive insight into the therapeutic potential of the newly isolated compound **1**, a comparative in silico database was constructed using three well-known structurally related plant flavonoids—Quercitrin, Hyperoside, and Isoquercitrin—alongside the clinical drug Acarbose. The molecular docking simulations revealed a highly favorable energy trend, wherein Compound **1** exhibited the highest predicted binding affinity (−9.5 kcal/mol), systematically outperforming Quercitrin (−9.3 kcal/mol), Hyperoside (−8.3 kcal/mol), Isoquercitrin (−7.9 kcal/mol), and Acarbose (−7.2 kcal/mol) (Table 2).

**Figure 3:**
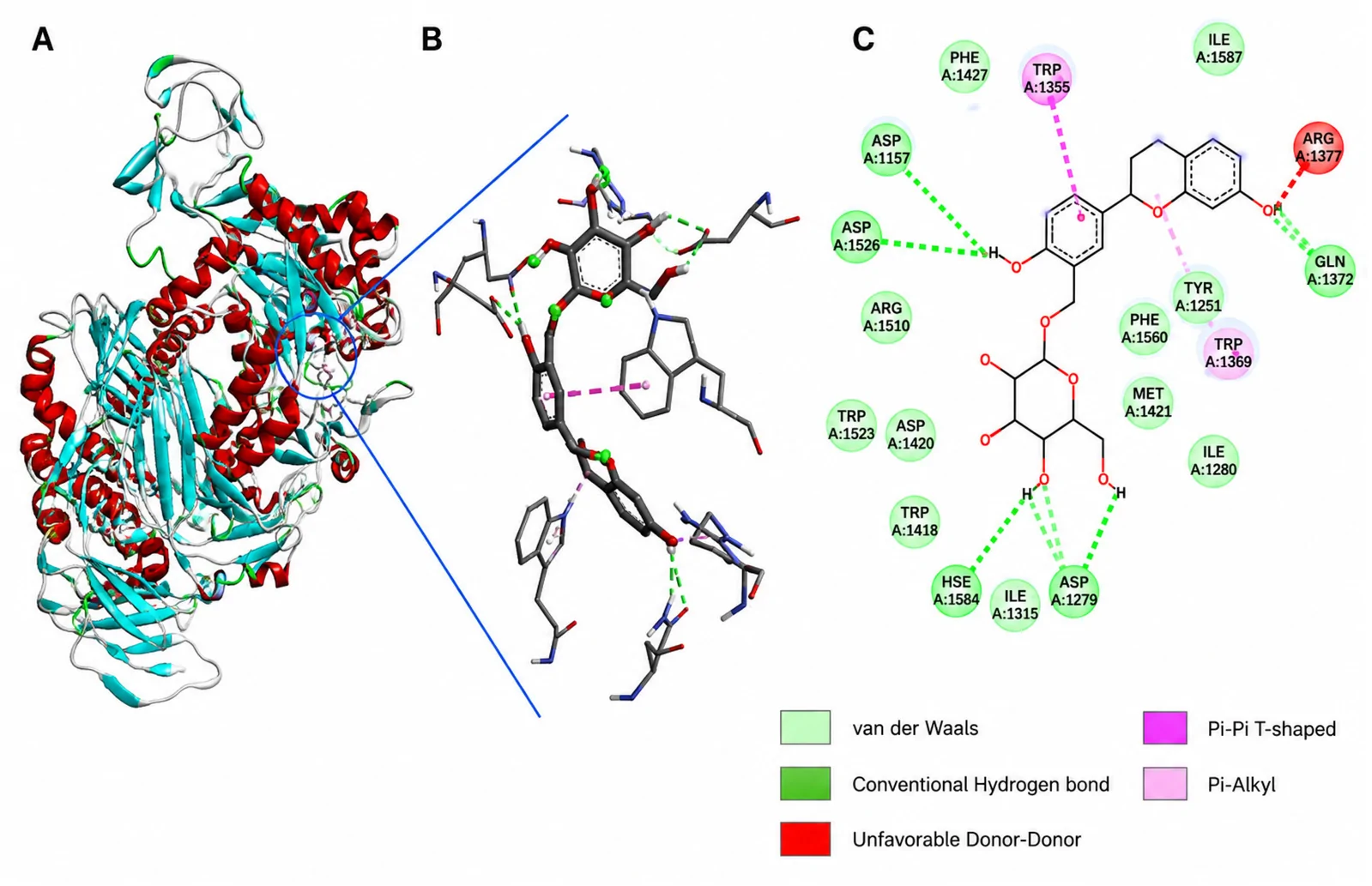
Molecular docking using the SeamDock web server (univ-paris-ditergent.fr) for compound **1** with HMG (PDB ID: 3TOP). (**A**) 3D view of how **1** fit in HMG’s active site. (**B**) Zoomed-in look at **1**’s orientation inside the active site. (**C**) 2D interaction diagram showing the main interactions between **1** and the amino acids around HMG’s active site.

**Figure 4:**
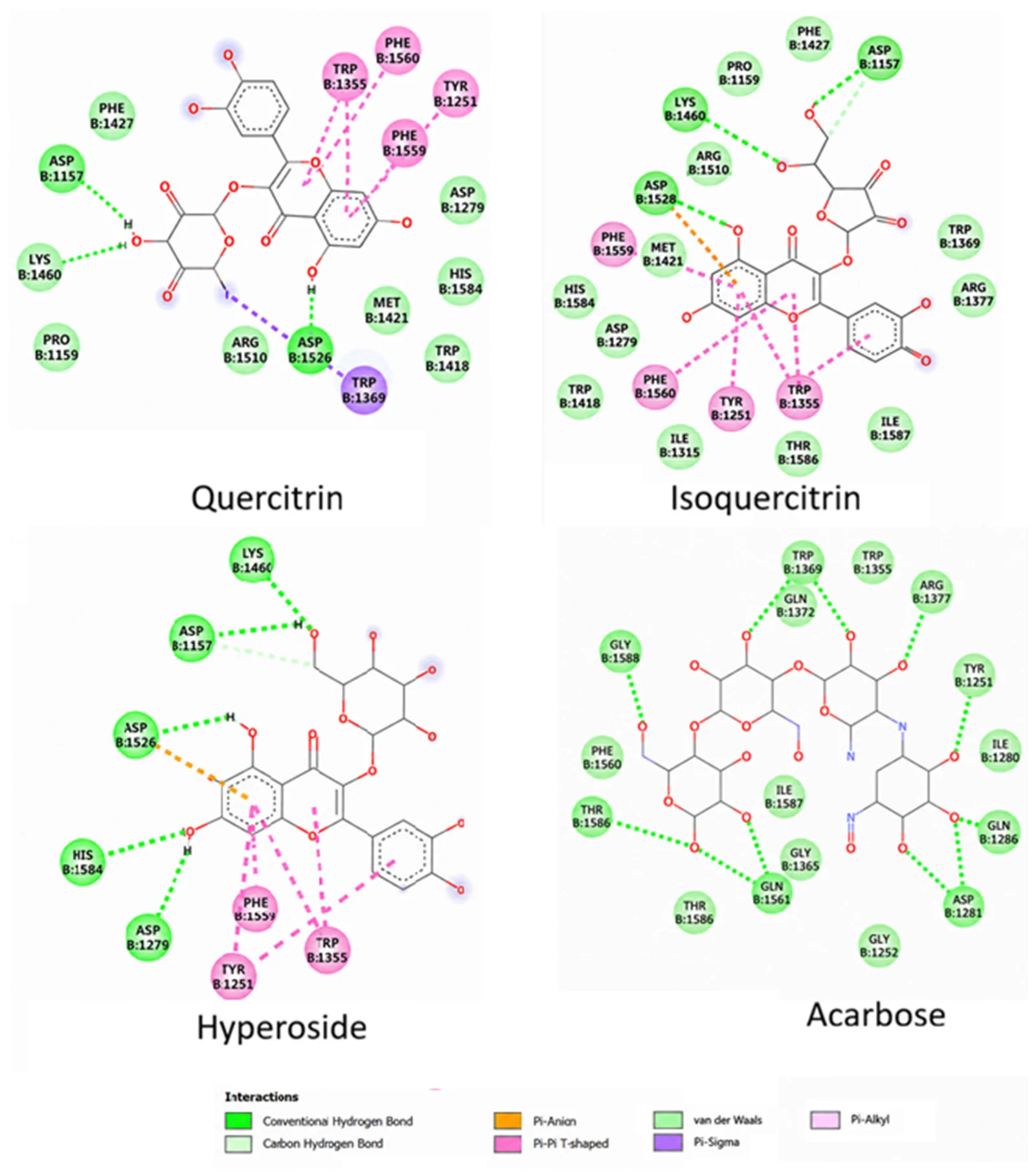
Two-dimensional interaction diagram showing the main interactions between Quercitrin, Isoquercitrin, Hyperoside, Acarbose, and the amino acid residues around the HMG active site.

Structurally, this energy hierarchy can be rationalized by the distinct spatial conformations of the ligands within the human intestinal maltase-glucoamylase (MGAM, PDB ID: 3TOP) pocket. While Quercitrin closely approaches the affinity of Compound **1** due to its similar hydrophobic interactions, the unique spatial positioning of the rhamnose/galactose moieties in Hyperoside and Isoquercitrin appears to induce minor steric constraints within the enzymatic cavity, slightly lowering their binding efficiency. Most notably, the non-sugar flavan framework of Compound **1** coupled with its β-D-glucoside group yields a superior topological fit over the highly flexible oligosaccharide structure of Acarbose. This structural rigidity minimizes conformational entropy loss upon binding, allowing Compound **1** to anchor more deeply and stably into the catalytic pocket. These comparative findings strongly support the structural hypothesis that the newly isolated 4′,7-dihydroxyflavan-3′-O-β-D-glucoside framework represents a highly robust natural scaffold for α-glucosidase inhibition. The specific three-dimensional (3D) binding orientation and detailed intermolecular interactions of the newly isolated Compound **1** within the active site cavity of human intestinal maltase-glucoamylase (PDB ID: 3TOP) are explicitly depicted in Figure 3. To evaluate the structural variations in binding modes, the two-dimensional (2D) interaction maps benchmarking the reference drug Acarbose against the three structurally related plant flavonoids—Quercitrin, Isoquercitrin, and Hyperoside—are comparatively illustrated in Figure 4.

Crucially, the hydrophilic anchoring of **1** targets the exact catalytic center of human maltase-glucoamylase (PDB ID: 3TOP). In glycoside hydrolase family 31 (GH31) enzymes, conserved aspartic acid residues are the absolute catalytic drivers, serving as the nucleophiles and acid/base catalysts responsible for cleaving carbohydrate chains. The formation of conventional hydrogen bonds between **1** and key active site residues—namely Asp1157, Asp1526, Gln1372, and notably the catalytic driver Asp1279—confirms that the molecule effectively mimics and outcompetes natural substrate stabilization. Although an unfavorable donor–donor interaction was identified involving the Arg1377 residue, inducing a localized electrostatic strain, this minor repulsive penalty is structurally and energetically overridden by the surrounding cooperative network. Specifically, the directional stability provided by the core hydrogen bonds is strongly reinforced by an extensive hydrophobic array, including π-π T-shaped and π-alkyl interactions with Phe1560, Tyr1251, and Trp1355, which collectively stabilize the ligand–receptor complex. These non-polar engagements may restrict the conformational mobility of the active site throat, potentially obstructing the entry of natural starch substrates [23].

Collectively, the distinct intermolecular crosslinks and favorable binding energy observed in this study confirm that 4′,7-dihydroxyflavan-3′-*O*-*β*-D-glucoside possesses the structural requirements to serve as a highly potent natural competitive scaffold for future anti-hyperglycemic drug design [24]. In summary, the comparative molecular docking simulations demonstrated that the newly isolated Compound **1** features an exceptional structural fit and superior thermodynamic stability within the catalytic pocket of human intestinal maltase-glucoamylase, outperforming both the reference flavonoids and Acarbose. However, while high binding affinity confirms its potent pharmacodynamic potential at the target site, the clinical viability of a drug candidate is equally dictated by its physiological behavior inside the human body. Therefore, to ensure that this high binding efficacy translates into a safe and localized therapeutic effect within the gastrointestinal tract, the subsequent section systematically evaluates and compares the pharmacokinetic and toxicological (ADMET) profiles of these compounds.

### 3.3. ADMET Prediction of ***1***

The comparative ADMET profiling, as summarized in Table 2, systematically evaluates the pharmacokinetic behavior and systemic safety of the newly isolated Compound **1** alongside three well-known benchmark flavonoid glycosides (Quercitrin, Isoquercitrin, and Hyperoside) and the clinical control drug Acarbose. Intestinal absorption of oral therapeutics is heavily dictated by the molecule’s capability to cross epithelial barriers. Compound **1** exhibited a low Caco-2 permeability of −6.432 log cm/s (ideal threshold > −5.15 log cm/s) and a low Human Intestinal Absorption probability (HIA = 0.120), collectively demonstrating restricted passive transcellular diffusion. Crucially, Acarbose exhibits an even lower Caco-2 permeability of −7.082 and a high HIA probability of 0.999, which perfectly aligns with its established clinical role as a locally acting, non-absorbed intestinal luminal agent [25]. This shared pharmacokinetic trait is highly desirable for α-glucosidase inhibitors targeting dietary carbohydrate digestion. Because the therapeutic molecular targets (MGAM) reside exclusively within the brush border membrane of the small intestine, poor systemic absorption prevents premature drug clearance and ensures that Compound **1** remains concentrated locally within the gut lumen to exert its competitive enzyme inhibition. Interestingly, while Acarbose-like luminal action is anticipated, Compound **1** is predicted to be neither a P-gp inhibitor (0.000) nor a major substrate (0.224). In comparison, Quercitrin displayed a high probability of being a P-gp substrate (0.567), which may accelerate its efflux clearance back into the lumen via active transport, whereas Compound **1**’s retention is governed primarily by its passive physicochemical hydrophilicity.

**Figure 5:**
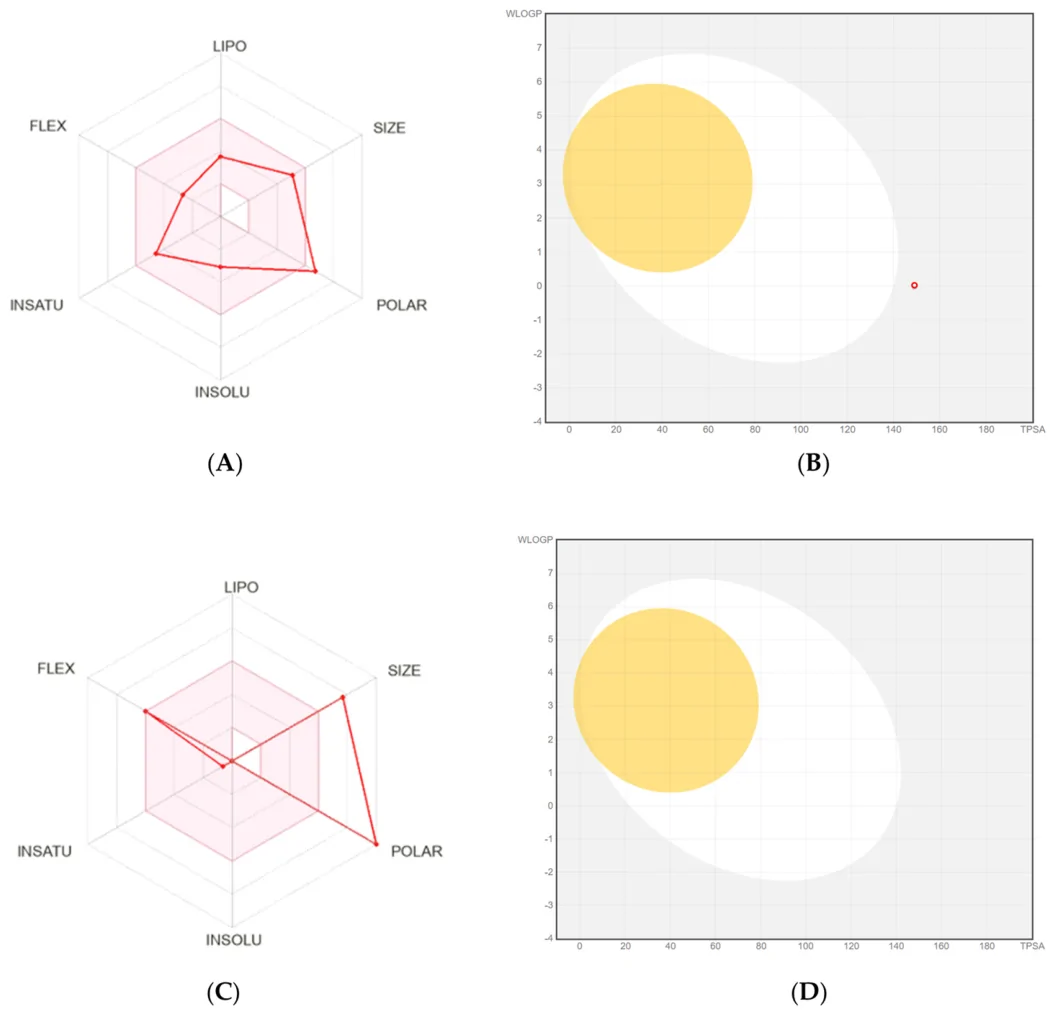
Radar bioavailability and BOILED-Egg of compound **1** (**A**,**B**) and acarbose (**C**,**D**) obtained from SwissADME.

To visually map the physicochemical descriptors driving this localized action, Bioavailability Radar and BOILED-Egg analyses were implemented via the SwissADME platform (Figure 5). In the development of conventional drugs targeting systemic organs, extreme deviations in the radar diagram—especially observed in Acarbose—would immediately eliminate a compound from the drug discovery pipeline due to anticipated poor absorption. However, in the context of Type 2 Diabetes Mellitus therapy via glycoside hydrolase inhibition, this radar distortion highlights a unique structural asset. The clear distortion in the POLAR parameter for both molecules is due to their strong hydrophilic properties, caused by a Topological Polar Surface Area (tPSA) of 149.07 Å^2^ for Compound **1** and an extreme 325.75 Å^2^ for the complex oligosaccharide structure of Acarbose. The presence of an oxygen-rich glycon moiety (β-D-glucose) and surrounding hydroxyl groups significantly increases polar charge density, pulling the geometric profile outside the ideal threshold (140 Å^2^). This intense hydrophilicity prevents the molecules from penetrating the lipid double layer of intestinal enterocytes via passive diffusion. Consequently, both Compound **1** and Acarbose are mathematically positioned in the outer gray region of the BOILED-Egg plot, confirming that they will remain unabsorbed and accumulate at high concentrations within the intestinal lumen. This extended residence time within the gut throat is vital to promote sustained competitive bonding with MGAM, effectively blocking the cleavage of dietary oligosaccharides into glucose and blunting postprandial glycemic spikes directly from within the gastrointestinal tract without requiring systemic entry.

In terms of metabolic and excretion kinetics, Compound **1** exhibits an ideal profile to mitigate the risk of clinical drug–drug interactions (DDIs). The molecule is predicted to act as neither a substrate (0.059) nor an inhibitor (0.004) of Cytochrome P450 3A4 (CYP3A4). Given that CYP3A4 is responsible for metabolizing the majority of commercial therapeutics, the non-interactive nature of Compound **1** minimizes the risk of dangerous serum spikes associated with polypharmacy regimens, while shielding the scaffold from extensive first-pass metabolism in the liver. In sharp contrast, Hyperoside presented a remarkably high risk of CYP3A4 inhibition (0.758), which could trigger severe toxicological complications when co-administered with other medications. Furthermore, the Human Liver Microsome (HLM) stability score (0.051) indicates that Compound **1** is highly stable against hepatic degradation. Its plasma clearance value of 2.502 mL/min/kg falls into the low-clearance category, reflecting a controlled and steady elimination rate by the clearance organs. This balanced excretion profile is coupled with a favorable plasma half-life (T_1/2_ = 3.119 h). This relatively short half-life span is highly advantageous in medicinal chemistry, ensuring that the compound provides an optimal therapeutic window without the risk of accumulation in systemic tissues that could trigger chronic toxic effects.

Toxicological screening unveiled a vital, comprehensive safety advantage for Compound **1** across all vital organ parameters. Under the medicinal chemistry filters, the compound recorded a low reactive score of 0.235 (Category 0), proving that the scaffold is chemically stable and carries a very low probability of forming non-specific covalent bonds with cellular macromolecules. This structural security is strongly reinforced by a score of 0 on the Acute Toxicity Rule, confirming that the molecule is free of 20 distinct classes of hazardous sub-structural fragments (toxicophores). Most notably, Compound **1** demonstrated an exceptional safety window regarding hepatotoxicity, exhibiting a remarkably low probability for Drug-Induced Liver Injury (DILI = 0.213). Conversely, Acarbose presented a critically high hepatotoxic risk (DILI = 0.882), which clinically correlates with the documented elevation of serum transaminase levels during chronic therapy [26,27]. Similarly, the reference flavonoids Quercitrin (0.733), Isoquercitrin (0.698), and Hyperoside (0.717) all heavily crossed the dangerous toxicological threshold (DILI > 0.50). On the hERG blocker parameter associated with cardiotoxicity, Compound **1** recorded a score of 0.399. While this value rests near the borderline zone, it remains safely below the danger threshold (0.50), confirming it is a non-blocker that carries no substantial risk of triggering QT interval prolongation or fatal cardiac arrhythmias. Ultimately, the integration of these comparative pharmacodynamic and pharmacokinetic metrics establishes Compound **1** as a highly promising natural scaffold, balancing optimized local luminal retention, metabolic stability, and an exceptional safety profile that overcomes the toxicological limits of current options.

An acknowledgeable limitation of the present study is the absence of immediate in vitro or in vivo experimental validation for the predicted biological activity of compound **1**. While the newly isolated scaffold was successfully characterized via comprehensive NMR analysis, its inhibitory potential against human maltase-glucoamylase relies strictly on rigorous in silico docking and atomistic simulations. Computational modeling serves as a highly efficient screening springboard; however, it cannot fully replicate the complex biochemical kinetics of a living or cell-free enzymatic environment. To establish a definitive pharmacological profile, quantitative benchmarking through in vitro enzymatic assays and subsequent cellular evaluation represent the mandatory next milestones in our ongoing research timeline.

## 4. Materials and Methods

### 4.1. Plant Material

The stem bark of *A. saponaria* plant had been taken from Ternate, North Maluku and determined at Biology Department, Institute of Technology Bandung.

### 4.2. Chemicals, Chromatography, and Spectroscopy Instruments

The reversed-phase analytical high-performance liquid chromatography (HPLC) used is a Dionex Liquid Chromatography with an automatic sample injector ASI-100, Alltech Evaporative Light Scattering Detector ELSD-800, and Foxy Jr. sample collector. The column used is a reversed-phase Phenomenex Prodigy C18 5-ODS column (5 µm, 250 × 4.6 mm), with the column temperature set at 40 °C using a Thermostated Column Compartment TCC-100. The mobile phase started with 10% acetonitrile (CH_3_CN) in H_2_O (0.05% TFA) for 2 min in isocratic elution, then a gradient elution to 50% CH_3_CN-H_2_O (0.05% TFA) over 24 min, followed by 10 min of isocratic elution, then a gradient to 100% CH_3_CN over 2 min, and finally 4 min of isocratic elution. The flow rate was 1 mL/min.

One- and two-dimensional nuclear magnetic resonance spectra were measured on a Varian UNITY INOVA-500 NMR spectrometer, where the proton (^1^H) was at 500 MHz and the ^13^C was at 125 MHz in MeOD solvent. The spectra were recorded in a 3 mm diameter tube at a temperature of 23 °C. The two-dimensional NMR techniques are HH-COSY (Correlation Spectroscopy), HSQC-DEPT (Heteronuclear Single Quantum Correlation-Distortionless Enhanced Proton Test), and CIGAR (Constant time Inverse detected Gradient Accordion Rescaled long-range heteronuclear multiple bond correlation). The mass spectrum was measured on an LCT Micro mass Liquid Chromatography Mass Spectrometry (LC-MS) using positive Electrospray Ionization (ESI) at 3200 V and a temperature of 150 °C.

### 4.3. Extraction and Isolation

*A. saponaria* fresh dark as much as 2400 g cleaned, peeled the husk, and then cut into thin slices with a stainless-steel blade, dried at room temperature in an open room and assisted with the fan until the moisture content approximately 10%. Results of drying get dried samples of bark as much as 1100 g. The dried sample then ground in a blender until pumpkin fineness of 100 mesh.

A total of 1100 g of dried *A. saponaria* stem bark powder was extracted using petroleum ether (b.p. 60–80 °C; 3 × 6.5 L) to remove lipids. After each extraction phase, the extracts were allowed to stand for 24 h at room temperature, the filtrate was collected and combined, and then the last filtrate was tested with parchment paper until no stain appeared, indicating that all the lipids had been extracted. The residue was re-macerated with methanol (3 × 6.2 L), filtered, and then the filtrates combined (the final filtrate was tested by TLC until no stain appeared). The methanol extract filtrate was evaporated to produce 157 g of condensed methanol and transferred into a glass bottle, sealed, and stored in the refrigerator. Furthermore, 50 g of the methanol extract was dissolved in water and partitioned with n-butanol (1:1 *v*/*v*, fifteen times). The butanol fraction was collected and evaporated to dryness / under reduced pressure, yielding 48.73 g of a viscous residue. A 20 g portion of this concentrated fraction was dissolved in methanol (150 mL), and an excess of diethyl ether (250 mL) was added to induce precipitation. The mixture was filtered to obtain a brown precipitate (9.63 g), hereafter referred to as the purified butanol fraction. A total of 2.67 g of the butanol extract was subjected to gel filtration chromatography on Sephadex LH-20 (2.0 × 50 cm; 100 g) using methanol as the eluent, yielding 60 eluent bottles (10 mL each). Each eluate was grouped into eight fractions (Fraction S1-S8) based on the analytical HPLC profile. Fraction S4 (153 mg) was further purified using semi-preparative reversed-phase HPLC to produce five peaks with respective retention times (t_R_): peak 1, t_R_ 10.25; peak 2, t_R_ 15.42; peak 3, t_R_ 16.20; peak 4, t_R_ 17.35; and peak 5, t_R_ 19.05 min. The fourth peak, labeled as **1**, weighed 1.8 mg.

### 4.4. Ligand and Protein Preparation for Molecular Docking

#### 4.4.1. Macromolecule Preparation

The three-dimensional crystal structure of human intestinal maltase-glucoamylase (MGAM) was retrieved from the RCSB Protein Data Bank (PDB) using the PDB ID: 3TOP [14]. Macromolecule preparation was conducted using BIOVIA Discovery Studio Visualizer [28] and the SeamDock platform wizard [29]. All heteroatoms, including crystallographic water molecules H_2_O and co-crystallized solvent molecules, were carefully removed from the protein structure to eliminate potential interference with ligand binding. Polar hydrogen atoms were added to the protein, and Kollman charges were assigned to satisfy the valency requirements of the amino acid residues. The structure was saved in the standard PDBQT format for subsequent docking simulations.

#### 4.4.2. Ligand Preparation and Energy Minimization

The structure of the newly isolated flavonoid glycoside, 4′,7-dihydroxyflavan-3′-*O*-*β*-D-glucoside (**1**), alongside the reference inhibitor Acarbose and comparative flavonoids (Quercitrin, Isoquercitrin, and Hyperoside), were sketched using ChemDraw software (version 20.0). The two-dimensional (2D) structures were converted into three-dimensional (3D) spatial conformations. Energy minimization of all ligands was performed using the MM2 force field to optimize the geometric coordinates and attain the global minimum energy state. All rotatable bonds within the ligands were allowed to remain flexible during the process. The optimized structures were then converted into PDBQT format, with Gasteiger partial charges assigned to all atoms.

#### 4.4.3. Docking Protocol and Grid Box Setup

Flexible molecular docking simulations were executed using the SeamDock web server, which integrates the AutoDock docking engine using Seamdock software (version 1.0, updated May 2021). To ensure a targeted simulation, the grid box was centered around the binding pocket defined by the native co-crystallized ligand (Acarbose) within the 3TOP structure. The grid coordinates were specified with a spacing of 0.553 Å, and the box dimensions were set to encompass the critical catalytic drivers and surrounding active site residues, specifically Arg1377, Gln1372, Gly1365, and Asp1279. The precise grid center parameters were set at coordinates: X = −20, Y = −5, Z = −18 and dimensions of 49 Å × 67 Å × 53 Å. The conformation with the lowest semi-empirical binding free energy (kcal/mol) and the most favorable interaction network was selected for detailed biomolecular visualization.

### 4.5. ADMET Analysis

The ADMET properties and drug-likeness were evaluated by using ADMETlab 3.0, a and swissADME the free web-based tools. This platform integrates databases curated with high-quality experimental data, guaranteeing reliability and accuracy. Compounds were entered in SMILES notation to this platform to be analyzed. Several ADMET parameters have been selected, including Caco-2 permeability, P-gp inhibitor-substrate, Human Intestinal Absorption (HIA), 30% bioavailability, Plasma Protein Binding (PPB), volume of distribution (V_Dss_), Blood–Brain Barrier (BBB), CYP3A4 inhibitor-substrate, Human Liver Microsomal (HLM) stability, CLplasma, T_1/2_, hERG blockers, DILI, and acute oral toxicity in rats. These parameters were selected for the initial screening of the pharmacokinetic properties of the test compound.

## 5. Conclusions

In this study, a new flavonoid glycoside was successfully isolated for the first time from the stem bark of *A. saponaria* using a combination of gel filtration and reversed-phase high-performance liquid chromatography (RP-HPLC) techniques. Comprehensive structural elucidation utilizing mass spectrometry (MS) along with 1D and 2D NMR spectroscopy (^1^H, ^13^C, HSQC-DEPT, COSY, and CIGAR) identified the new compound as 4′,7-dihydroxyflavan-3′-*O*-*β*-D-glucoside (**1**). The biological and pharmacokinetic potential of Compound **1** was further evaluated through comparative in silico profiling against a database of known related plant flavonoids (Quercitrin, Hyperoside, and Isoquercitrin) and the standard clinical drug Acarbose. In computational molecular docking simulations against human intestinal maltase-glucoamylase (PDB ID: 3TOP), Compound **1** exhibited a superior predicted binding affinity of −9.5 kcal/mol, systematically outperforming Quercitrin (−9.3 kcal/mol), Hyperoside (−8.3 kcal/mol), Isoquercitrin (−7.9 kcal/mol), and Acarbose (−7.2 kcal/mol).

Molecular interaction mapping via BIOVIA Discovery Studio revealed that this strong affinity is driven by a highly stable network of conventional hydrogen bonds with crucial active site residues (Arg1377, Gln1372, and Gly1365), coupled with π-π T-shaped and π-alkyl hydrophobic forces that effectively anchor the molecule within the enzyme’s catalytic pocket, easily overriding a localized electrostatic strain at Asp1279. Furthermore, integrated ADMET modeling confirmed favorable local intestinal retention for the compound, characterized by low Caco-2 permeability (−6.432) and low human intestinal absorption (HIA = 0.120), which optimize its luminal action while restricting systemic exposure. Crucially, toxicological screening highlighted a profound safety profile for Compound **1** with a very low hepatotoxic probability (DILI = 0.213), significantly outperforming the high-risk profiles of Acarbose (DILI = 0.882) and the reference flavonoids (DILI > 0.69).

Taken together, these predictive findings demonstrate that Compound **1** represents a highly promising natural competitive scaffold for the development of potent α-glucosidase inhibitors. While the present study establishes a robust computational foundation, subsequent wet-lab in vitro and in vivo functional assays are warranted in future investigations to definitively validate its enzyme inhibitory kinetics. Consequently, this novel scaffold serves as a highly feasible candidate to be accommodated into future laboratory synthesis and biological testing phases.

## Figures and Tables

**Table 1:** ^1^H-NMR and ^13^C-NMR chemical shifts for **1** from the stem bark of *A. saponaria*.

C and H Positions	δ C	δH (ppm), Multiplicities, *J* (Hz)
Flavan core		
2	78.4	4.97 (*dd*; 1.0; 8.5)
3	30.9	1.97 2.13
4	25.0	2.63 2.82
4a	114.7	-
5	130.9	6.85 (*d*; 8.3)
6	109.3	6.31 (*d*; 8.3)
7	157.93	-
8	104.0	6.27 (s)
8a	157.90	-
1′	140.0	-
2′	118.5	7.30 (s)
3′	149.3	-
4′	122.4	7.07 (*d*; 8.1)
5′	120.6	7.24 (*d*; 8.1)
6′	149.1	-
Sugar unit		
1″	104.1	4.86 (*d*; 7.7)
2″	75.1	3.48 (*dd*; 7.7; 11.9)
3″	77.8	3.49 (*dd*; 9.8; 11.9)
4″	71.4	3.36 (m)
5″	78.2	3.34 (m)
6″	62.4	3.67 (m) 3.82 (m)

**Table 2:** Integrated In Silico Binding Affinity and ADMET Comparative Profiling of Compound **1** and Reference Flavonoid Glycosides.

Category/Parameter	Compound 1	Quercitrin	Hyperoside	Isoquercitrin	Acarbose
**Pharmacodynamics (Docking)**					
Predicted Binding Affinity (kcal/mol)	**−9.5**	**−9.3**	**−8.3**	**−7.9**	**−7.2**
**Absorption & Permeability**					
Caco-2 Permeability (log *P_app_*)	−6.432	−6.176	−6.018	−6.260	−7.082
HIA (Human Intestinal Absorption)	0.120	0.263	0.232	0.124	0.999
P-gp Inhibitor (Probability)	0.000	0.000	0.000	0.000	0.000
P-gp Substrate (Probability)	0.224	0.567	0.132	0.154	1.000
**Distribution**					
PPB (Plasma Protein Binding, %)	88.320	85.726	84.415	85.252	14.378
*V_Dss_* (Volume of Distribution, L/kg)	0.040	−0.086	−0.130	−0.005	−5.170
BBB Penetration (Probability)	0.363	0.000	0.000	0.001	0.000
**Metabolism & Excretion**					
CYP3A4 Inhibitor (Probability)	0.004	0.138	0.758	0.058	0.000
CYP3A4 Substrate (Probability)	0.059	0.000	0.000	0.000	0.000
HLM Stability (Probability)	0.051	0.668	0.904	0.732	0.038
Clearance-plasma (mL/min/kg)	2.502	4.283	5.494	5.571	0.115
Half-life (T_1/2_, h)	3.119	2.842	2.460	2.304	3.599
**Toxicity Profiles**					
hERG Blocker (Probability)	0.101	0.044	0.025	0.017	0.001
hERG Blocker (10 μM)	0.399	0.585	0.336	0.287	0.017
DILI (Drug-Induced Liver Injury)	0.213	0.733	0.717	0.698	0.882
Rat Oral Acute Toxicity	0.109	0.430	0.199	0.071	0.001

## Data Availability

The original contributions presented in this study are included in the article/Supplementary Material. Further inquiries can be directed to E.J.P as the corresponding author.

## Supplementary Materials

The following supporting information can be downloaded at: https://www.mdpi.com/article/10.3390/ph19091391/s1, Figure S1: ^1^H-NMR spectrum of Compound **1**, expanded at the aromatic area (Varian Inova, 500 MHz; CD3OD). Figure S2: ^1^H-NMR spectrum of compound **1**, expanded at the chemical shift γ 4,00 – 6,00 ppm (Varian Inova, 500 MHz; CD3OD). Figure S3: ^1^H-NMR spectrum of compound **1**, expanded at the chemical shift γ 3,10 – 3,90 ppm (Varian Inova, 500 MHz; CD3OD). Figure S4: ^1^H-NMR spectrum of compound **1**, expanded at the chemical shift γ 1,00 – 3,00 ppm (Varian Inova, 500 MHz; CD3OD). Figure S5: ^13^C-NMR spectrum of compound **1** (Varian Inova, 125 MHz; CD3OD). Figure S6: HSQC-DEPT spectrum of compound **1**. Figure S7: ROESY spectrum of compound **1**. Figure S8: CIGAR spectrum of compound **1**.

## Abbreviations

The following abbreviations are used in this manuscript:

ADMET	Adsorption, distribution, metabolism, excretion, toxicity
ARG	Arginine
BBB	Blood–Brain Barrier
DILI	Drug-induced liver injury
DM	Diabetes Mellitus
F-30	Bioavailability above 30%
FLEX	Flexibility
GLN	Glutamine
GLY	Glisin
HIA	Human Intestinal Absorption
HLM	Human liver microsomal
INSATU	Insaturation
INSOLU	Insolubility
LIPO	Lipophility
Log S	Solubility (logarithm of aqueous solubility)
P-gp	P-glycoprotein
PPB	Plasma protein binding
POLAR	Polarity
SIZE	Size of molecule or molecular weigh
SMILES	Simplified Molecular Input Line Entry System
T_1/2_	Elimination half-life
VDss	Volume distribution
