# Supplementary material for: A New Flavonoid Glycoside from the Stem Bark of Albizia saponaria: Isolation, Structural Elucidation, and In Silico Evaluation as a Potent α-Glucosidase Inhibitor
Source: Pharmaceuticals (Basel). 2026 Sep 2;19(9):1391. doi: 10.3390/ph19091391 (PMC13610131; doi:10.3390/ph19091391)
Supplement: Supplementary file 1 [file pharmaceuticals-19-01391-s001.zip › pharmaceuticals-4461405-supplementary.pdf]

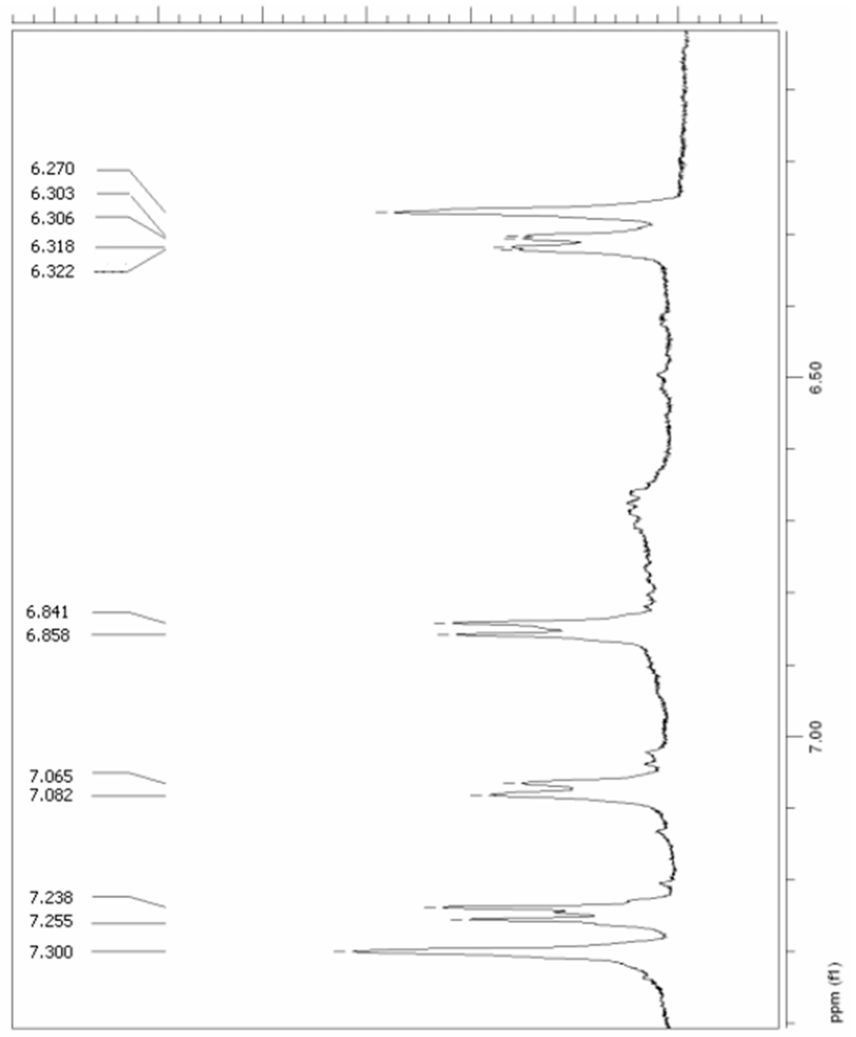

Figure S1: ^{1}H-NMR spectrum of Compound **1**, expanded at the aromatic area (Varian Inova, 500 MHz; CD3OD).

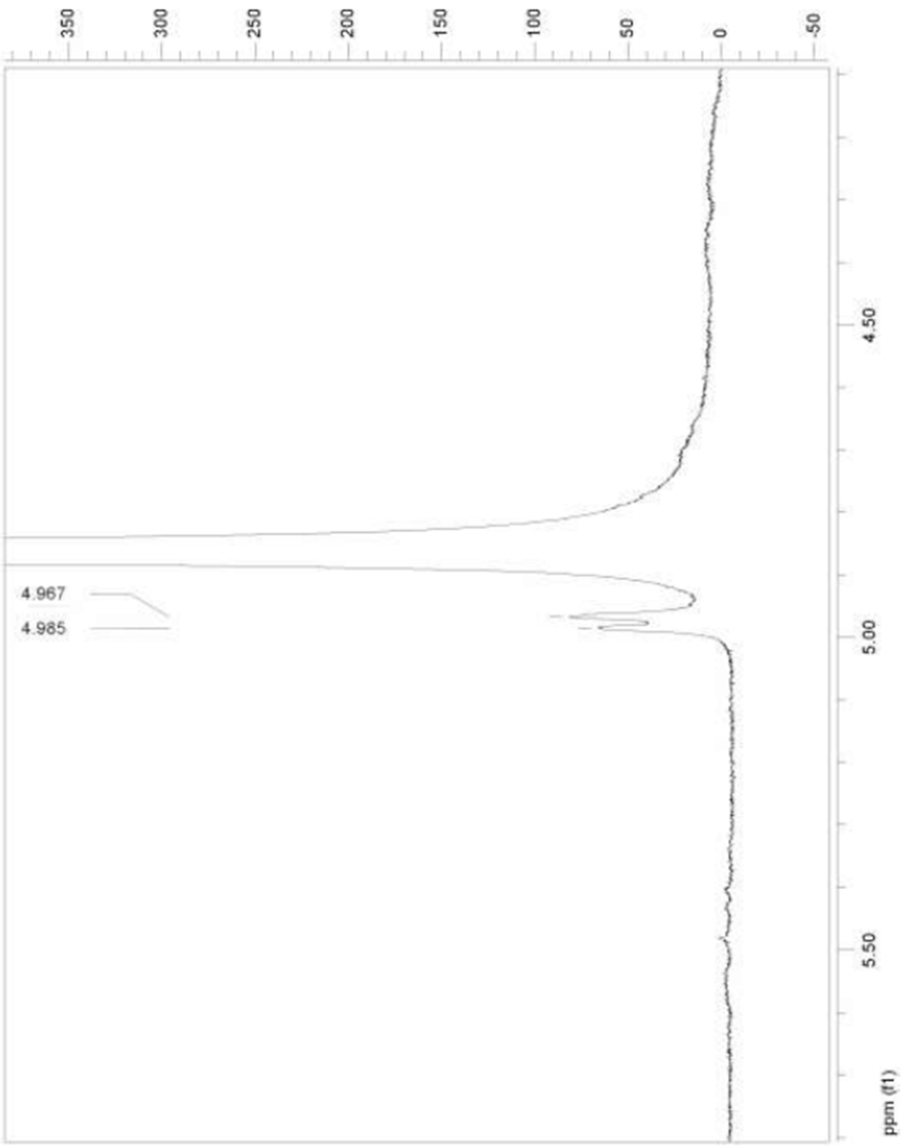

Figure S2: ^{1}H-NMR spectrum of compound **1**, expanded at the chemical shift γ 4,00 – 6,00 ppm (Varian Inova, 500 MHz; CD3OD).

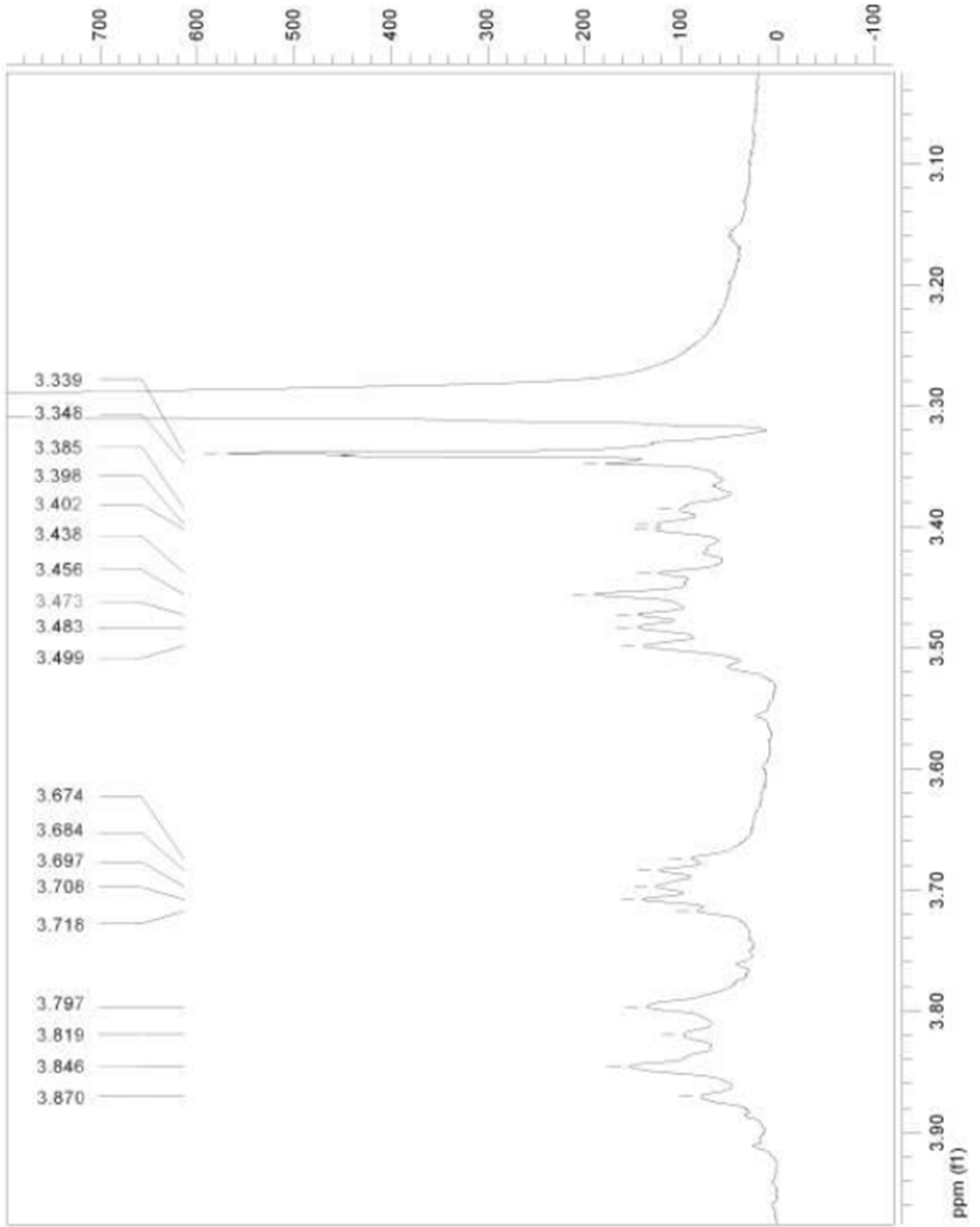

Figure S3: ^{1}H-NMR spectrum of compound **1**, expanded at the chemical shift γ 3,10 – 3,90 ppm (Varian Inova, 500 MHz; CD3OD).

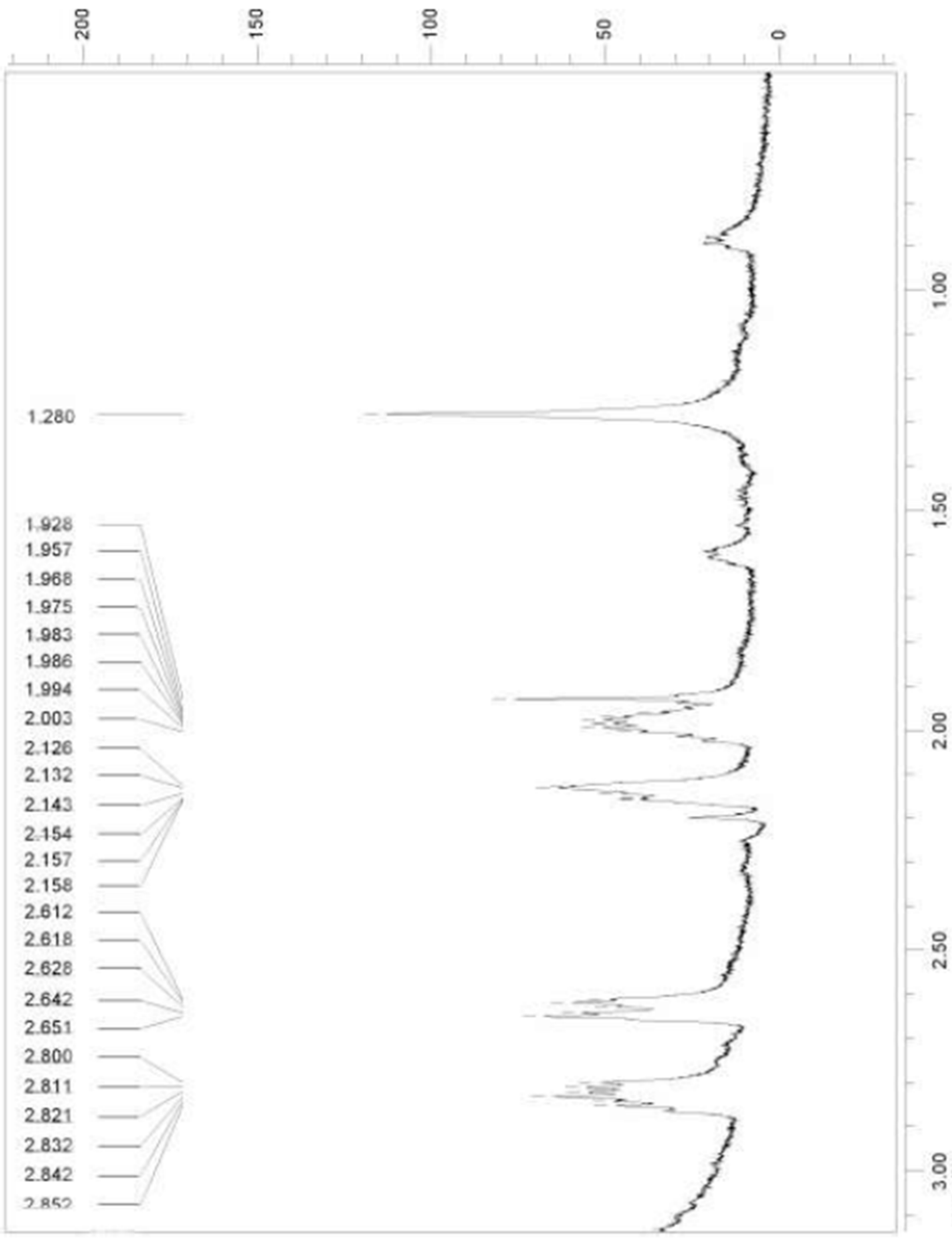

Figure S4: ^{1}H-NMR spectrum of compound **1**, expanded at the chemical shift γ 1,00 – 3,00 ppm (Varian Inova, 500 MHz; CD3OD).

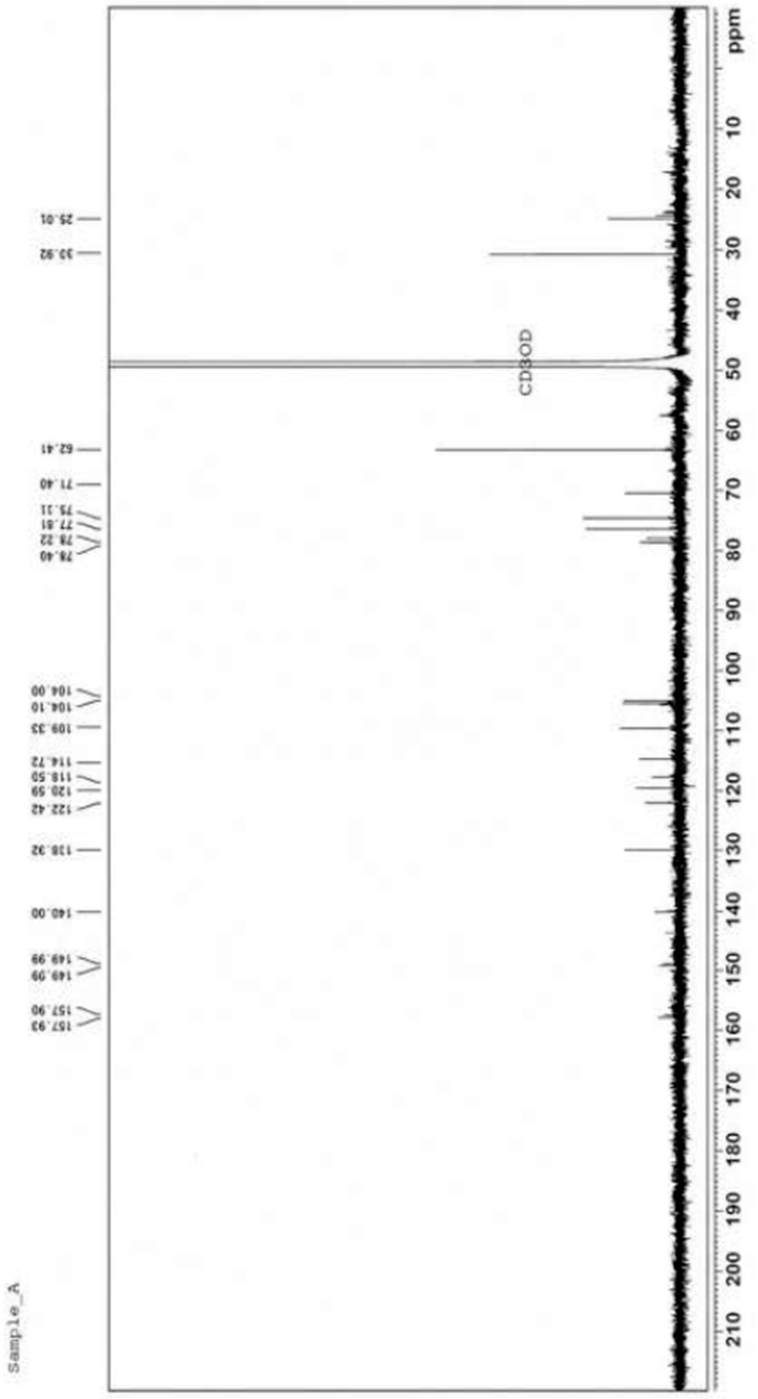

Figure S5: ^{13}C-NMR spectrum of compound **1** (Varian Inova, 125 MHz; CD3OD).

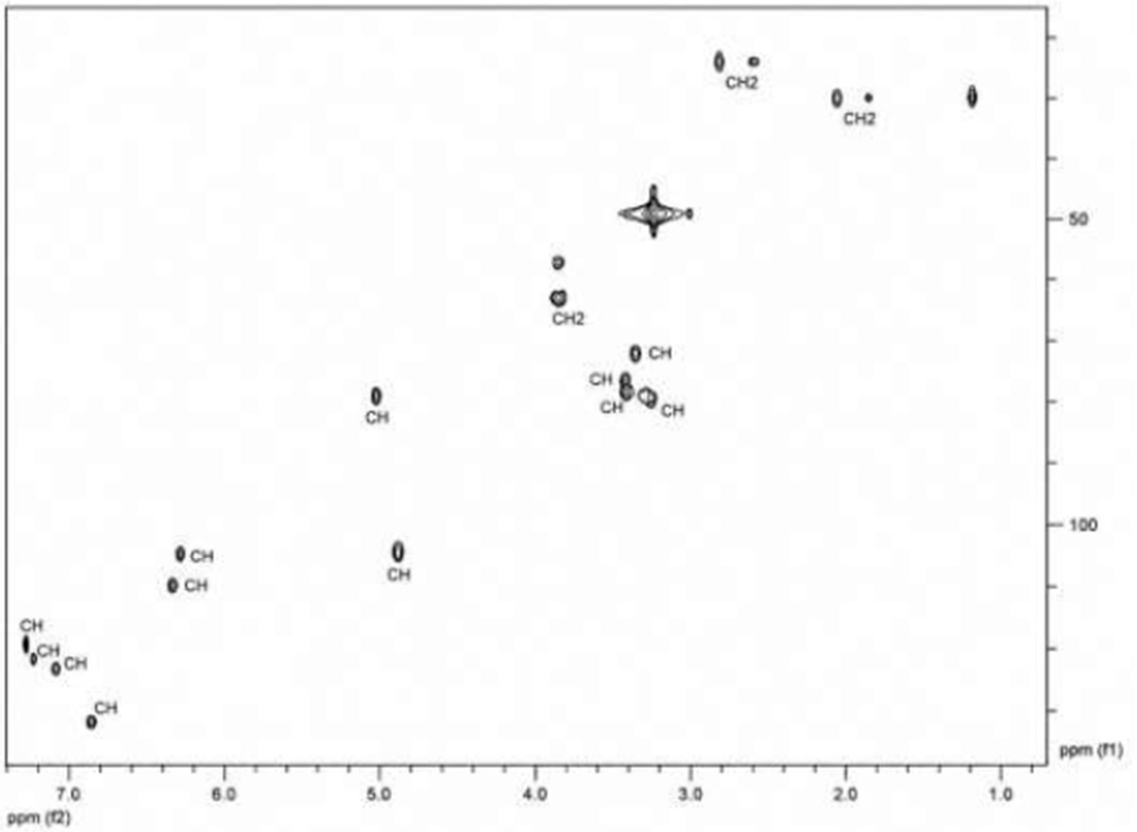

Figure S6: HSQC-DEPT spectrum of compound **1**.

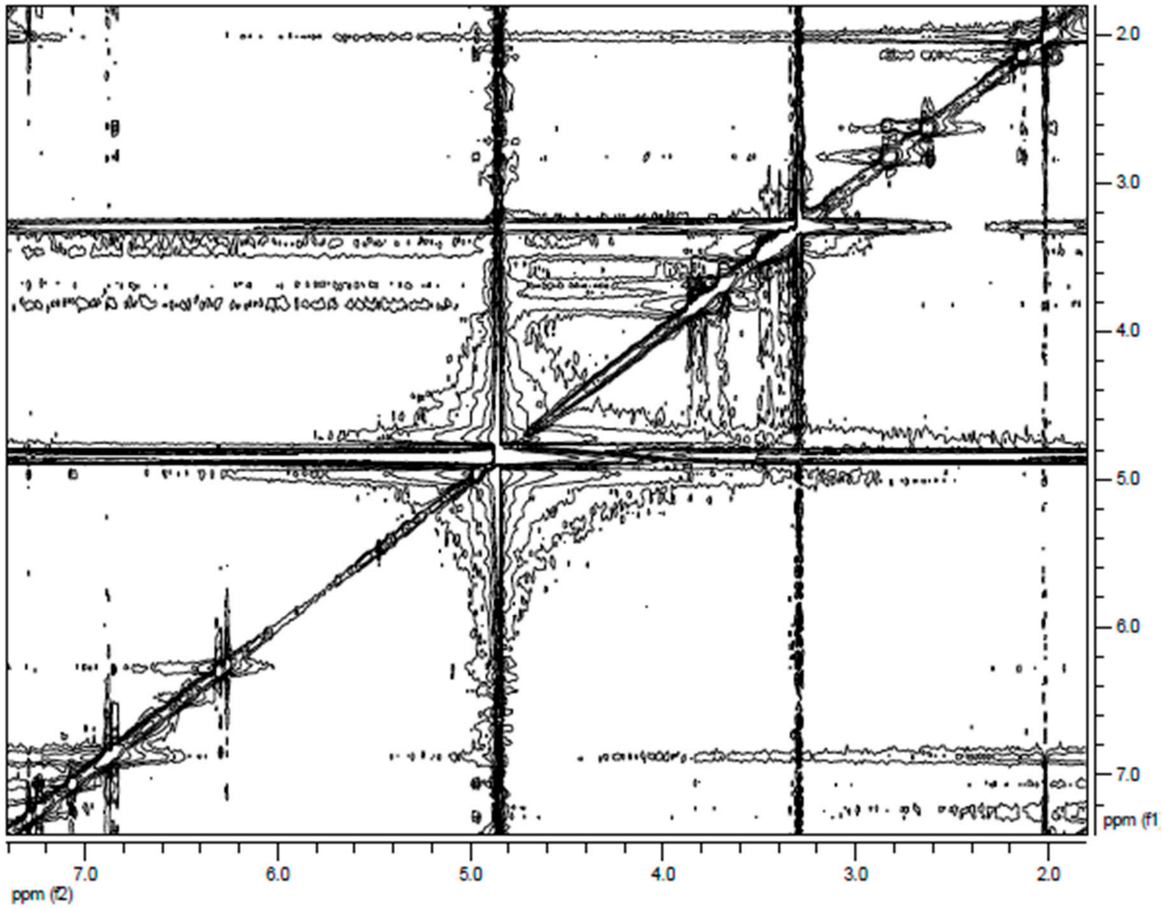

Figure S7: ROESY spectrum of compound **1**.

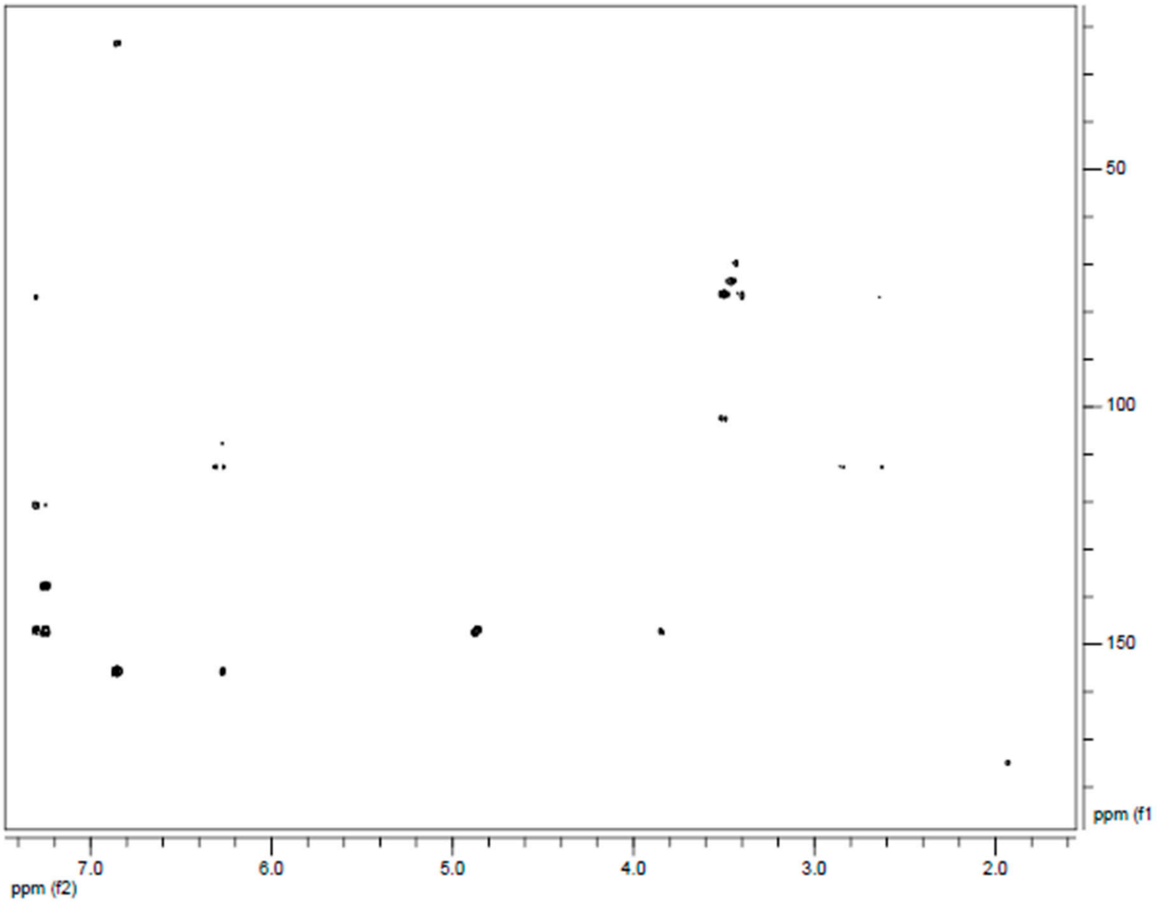

Figure S8: CIGAR spectrum of compound **1**.
